# Extracorporeal carbon dioxide removal integrated into continuous renal replacement therapy in pediatric patients with ARDS: a retrospective single-center case series

**DOI:** 10.1186/s12887-026-07656-y

**Published:** 2026-09-09

**Authors:** Martin Kuntz, Daniel Matheisl, Isabel Schüle, Daniel Klotz, Hans Fuchs, Hendryk Schneider

**Affiliations:** 1 Center for Pediatrics, Division of Neonatology and Pediatric Intensive Care Medicine, Faculty of Medicine, Medical Center - University of Freiburg, University of Freiburg, Breisacher Str. 62, 79106 Freiburg, Germany; 2 Center for Pediatric, Division of Pediatric Nephrology, Faculty of Medicine, Medical Center - University of Freiburg, University of Freiburg, Freiburg, Germany; 3 Department of Neonatology and Pediatric Intensive Care Medicine, Bethel Center for Pediatrics, University Hospital OWL, University of Bielefeld, Germany, Bielefeld, Germany

**Keywords:** Extracorporeal carbon dioxide removal, Continuous renal replacement therapy, Pediatric acute respiratory distress syndrome, Acute kidney injury, Pediatric critical care, Case series

## Abstract

**Background:**

Extracorporeal carbon dioxide removal (ECCO₂R) may facilitate lung-protective ventilation in children with pediatric acute respiratory distress syndrome (PARDS), but pediatric experience remains limited. We aimed to descriptively characterize the clinical use, physiological changes, technical implementation, and observed complications of ECCO₂R integrated into continuous renal replacement therapy (CRRT) in pediatric patients with PARDS and acute kidney injury (AKI).

**Methods:**

We conducted a retrospective, uncontrolled, single-center case series of pediatric patients with PARDS and AKI who received ECCO₂R integrated into an already indicated CRRT circuit between November 2023 and November 2025. To avoid non-independence of repeated observations, the first treatment episode per patient was included in the principal analysis (*n* = 7). Predefined physiological outcomes were assessed longitudinally at predefined time points. Longitudinal changes were analyzed using Friedman tests. Safety-related laboratory parameters were analyzed using Wilcoxon matched-pairs signed-rank tests.

**Results:**

Median age was 12 years (interquartile range [IQR] 6–14 years). ECCO₂R was delivered at a median blood flow of 270 mL/min (IQR 100–300), corresponding to 9% (IQR 6–20%) of the estimated cardiac output. Friedman analysis demonstrated significant longitudinal improvements in all predefined principal physiological outcomes, including arterial carbon dioxide tension (PaCO₂), peak inspiratory pressure (PIP), driving pressure (ΔP), and tidal volume (Vt) (all *P* < 0.05). Median absolute changes from baseline to 24 h were − 10 mmHg (IQR − 27 to − 3) for PaCO₂, − 11 mbar (IQR − 14 to − 4) for PIP, − 9 mbar (IQR − 18 to − 6) for ΔP, and − 1.6 mL/kg (IQR − 3.6 to − 0.3) for Vt. Four patients were successfully weaned from ECCO₂R. No predefined major bleeding, clinically apparent hemolysis, thromboembolic complications, or device-related infections attributable to ECCO₂R-CRRT were identified; circuit clotting requiring circuit exchange occurred in two patients.

**Conclusions:**

Integration of ECCO₂R into an already indicated CRRT circuit was technically feasible and temporally associated with reductions in PaCO₂ and ventilatory pressures in this cohort. Although these descriptive findings should be regarded as hypothesis-generating rather than evidence of treatment efficacy or device safety, they suggest that integrated ECCO₂R-CRRT may represent a pragmatic adjunctive support strategy in carefully selected pediatric patients requiring CRRT. Prospective multicenter studies are needed to better define patient selection, anticoagulation strategies, weaning protocols, and the role of integrated ECCO₂R-CRRT within pediatric extracorporeal respiratory support.

**Trial registration:**

German Clinical Trials Register (DRKS), DRKS00038799; retrospectively registered on April 23, 2026. https://drks.de/search/en/trial/DRKS00038799/details.

**Supplementary Information:**

The online version contains supplementary material available at https://doi.org/10.1186/s12887-026-07656-y.

## Highlights

ECCO₂R integrated into an already indicated CRRT circuit was technically feasible in carefully selected pediatric patients with PARDS and AKI.

Integrated ECCO₂R-CRRT was associated with rapid reductions in PaCO₂ and ventilatory pressures.

No predefined major bleeding, clinically apparent hemolysis, thromboembolic complications, or device-related infections attributable to ECCO₂R-CRRT were identified; circuit clotting requiring circuit exchange was the main observed complication.

This retrospective case series provides hypothesis-generating data to inform future prospective studies on patient selection, anticoagulation, and implementation of integrated ECCO₂R-CRRT.

**Supplementary Information:**

The online version contains supplementary material available at https://doi.org/10.1186/s12887-026-07656-y.

## Introduction

Acute respiratory distress syndrome (ARDS) and acute kidney injury (AKI) frequently coexist in critically ill children and are independently associated with increased morbidity and mortality, especially in the case of fluid overload, which independently increases mortality [1–3]. Mechanical ventilation itself contributes to renal injury through alterations in intrathoracic pressure, neurohormonal activation, and inflammatory mediator release, while AKI exacerbates pulmonary edema and acid–base disturbances, further complicating ventilatory management [4–6]. Consequently, simultaneous respiratory and renal support is a frequent challenge in pediatric intensive care.

Lung-protective ventilation using low tidal volumes while limiting inspiratory and driving pressures remains the cornerstone of ARDS management and the only intervention consistently associated with improved survival [7, 8]. However, escalation toward ultraprotective ventilation is frequently limited by hypercapnia and respiratory acidosis [9]. Extracorporeal carbon dioxide removal (ECCO₂R) selectively removes CO₂ from the blood, thereby decoupling carbon dioxide clearance from mechanical ventilation and enabling reductions in tidal volume, driving pressure, and minute ventilation without compromising gas exchange [10].

In adult patients, ECCO₂R has been evaluated in observational studies, randomized trials, and meta-analyses. A recent systematic review and meta-analysis by Stommel et al. reported consistent physiological reductions in arterial carbon dioxide tension (PaCO₂), tidal volume (Vt), peak inspiratory pressure (PIP), and driving pressure (ΔP) across heterogeneous populations with acute respiratory failure, including ARDS, although no mortality benefit was observed and complication rates varied by device type and blood-flow strategy [11]. Earlier analyses similarly reported reliable physiological effects but raised concerns regarding bleeding and device-related adverse events [12]. The REST trial, the largest randomized controlled trial to date, showed no mortality benefit of ECCO₂R-facilitated ultraprotective ventilation and reported increased rates of serious adverse events, particularly bleeding, highlighting the importance of careful patient selection [13].

Patients with combined ARDS and AKI represent a distinct subgroup in whom integration of ECCO₂R into an existing continuous renal replacement therapy (CRRT) circuit may be technically attractive. In contrast to venovenous extracorporeal membrane oxygenation (vvECMO), which requires high extracorporeal blood flows, large-bore cannulation, and a dedicated extracorporeal circuit to provide full gas-exchange support, ECCO₂R operates at substantially lower blood flows and provides partial respiratory support focused on CO₂ removal [14, 15]. When integrated into a CRRT platform, ECCO₂R can be delivered using an already existing extracorporeal circuit, avoiding additional vascular access and minimizing extracorporeal blood volume and circuit complexity. This combined ECCO₂R-CRRT approach is particularly attractive in patients who already require renal support [10, 16].

Certain pediatric populations are poor candidates for vvECMO despite severe respiratory failure. Children following hematopoietic stem cell transplantation (HCT), those with invasive fungal pulmonary infections, and patients with life-limiting underlying conditions often have a poor expected outcome or increased bleeding risk, rendering full extracorporeal life support a relative contraindication [17–23]. In these patients, particularly when ARDS is accompanied by AKI requiring CRRT, integration of low-flow venovenous ECCO₂R into an existing CRRT circuit represents a technically feasible alternative that avoids additional vascular access.

Despite growing adult experience, pediatric data on ECCO₂R remain extremely limited and are restricted to case reports and small case series, precluding robust conclusions regarding safety, efficacy, and patient selection [24–26]. Moreover, the multiECCO₂R gas-exchange membrane is approved for use in adult patients above a body weight of 40 kg only; its application in smaller pediatric patients represents a use outside the device´s approved labeling [27]. Given these knowledge gaps, systematic reporting of pediatric experience with ECCO₂R may help to better characterize patient selection, technical implementation, physiological changes, and observed complications.

Accordingly, the objective of this retrospective case series was to systematically report our initial institutional experience with ECCO₂R integrated into CRRT in pediatric patients, focusing on patient selection, technical implementation, physiological changes, and observed complications rather than treatment efficacy or device safety.

This case series is reported in accordance with the PROCESS 2025 Guideline (Preferred Reporting Of CasE Series) [28].

## Methods

### Study design

We conducted a retrospective, uncontrolled, single-center case series of pediatric patients who received extracorporeal carbon dioxide removal integrated into an existing continuous renal replacement therapy circuit (ECCO_2_R-CRRT). The case series included all patients treated with ECCO_2_R-CRRT using the Fresenius multiECCO_2_R blood gas exchanger between November 2023 and November 2025 at our tertiary pediatric intensive care unit (PICU).

This retrospective single-center case series was conducted at the PICU of the University Medical Center Freiburg and is reported in accordance with the PROCESS 2025 Guideline [28]. The retrospective study was approved by the Ethics Commission (IRB) of the Albert-Ludwigs-University Freiburg, Faculty of Medicine (Reference No. 26-1030-S1-retro; Date of approval March 30th, 2026), conducted in accordance with the Declaration of Helsinki, and retrospectively registered in the German Clinical Trials Register (DRKS00038799; Date of registration April 23rd, 2026) as retrospective observational study.

The multiECCO_2_R blood gas exchanger is intended for use in adults (> 18 years with body weight > 40 kg) in combination with renal replacement therapy using a CRRT platform. The described therapy in pediatric patients was approved on a case-by-case basis following multidisciplinary discussion (pediatric intensivist, pediatric nephrologist, and pediatric oncologist or neurologist) in accordance with institutional guidelines for emergency use. The patients and/or their legal guardians were informed and approved this use outside the currently approved indication.

### Data collection

Patient- and run-level data were collected by comprehensive review of the electronic medical records, intensive care documentation, and extracorporeal treatment records. Patient-level characteristics included age, body weight, underlying diagnosis, pediatric acute respiratory distress syndrome (PARDS) severity, number of ECCO₂R-CRRT treatment episodes, duration of extracorporeal support, and survival to PICU discharge. Run-level characteristics of the first ECCO₂R-CRRT treatment episode comprised vascular access site and catheter size, extracorporeal blood flow, sweep gas flow, anticoagulation strategy, successful discontinuation of ECCO₂R, and device-related complications, including membrane clotting requiring circuit exchange. All extracted data were reviewed for completeness and consistency before analysis.

Kidney function data were collected by the same comprehensive review of the electronic medical records and extracorporeal treatment documentation. The following variables were extracted: baseline serum creatinine (Cr_BASE_), serum creatinine at AKI diagnosis (Cr_AKI_), urine output during the 24 h preceding CRRT initiation, serum creatinine at CRRT initiation (Cr_CRRT_), percentage fluid overload (FO), the interval from PICU admission to fulfillment of KDIGO AKI criteria, and the interval from AKI diagnosis to CRRT initiation. Baseline body weight and baseline serum creatinine were defined as the most recent documented values before the onset of the critical illness. If unavailable, the admission body weight before substantial fluid resuscitation or fluid accumulation and the admission serum creatinine were used as surrogate baseline values. AKI was assessed retrospectively according to the KDIGO definition using serum creatinine, urine output, and the requirement for kidney replacement therapy [29, 30]. For descriptive purposes, KDIGO stages reported in Table 2 are based on serum creatinine criteria.

The principal physiological outcomes were longitudinal changes in PaCO₂, peak inspiratory pressure (PIP), driving pressure (ΔP), and tidal volume (Vt), reflecting the primary physiological objectives of ECCO₂R-CRRT. Exploratory physiological outcomes included pH, positive end-expiratory pressure (PEEP), oxygenation index (OI), ventilation index (VI), and vasoactive inotropic score (VIS). Outcome variables were recorded at the beginning of the therapy (baseline) and at 1, 6 ± 1 h, 12 ± 2 h, and 24 ± 2 h after initiation. To assess persistence of physiological effects beyond 24 h we included an analysis of the outcome values at a later time point than 24 h of therapy (∆T). Therefore, we analyzed the parameters 7 days after initiation of ECCO_2_R or within 24 h before discontinuation of ECCO_2_R, if therapy was shorter than 7 days.

Safety outcomes comprised laboratory markers associated with bleeding and hemolysis (including hemoglobin, platelet count, lactate dehydrogenase, and total bilirubin), red blood cell (RBC) and platelet transfusion requirements, and predefined adverse events.

Platelet and RBC transfusion requirements were assessed by comparing the mean number of transfusions per day before and after initiation of ECCO₂R-CRRT. To ensure comparable observation periods, the duration of each ECCO₂R-CRRT run was used to define the pre-treatment observation period. Thus, transfusion requirements during ECCO₂R-CRRT were compared with those during an equally long period immediately preceding ECCO₂R initiation. For treatment runs lasting longer than 7 days, transfusion requirements during the first 7 days of ECCO₂R-CRRT were compared with those during the 7 days before ECCO₂R initiation.

The calculation of OI, VI, and VIS was performed as follows:

$$\begin{aligned}\mathrm{OI}&=\left[\mathrm{FiO}_2*\mathrm{Paw}\left(\text{mean airway pressure}\right)\right.\\&\left.*100\right]/\mathrm{PaO}_{2}\:\mathrm{and}\end{aligned}$$

$$\begin{aligned}\mathrm{VI}&=\left[\mathrm{PaCO}_2*\:\mathrm{PIP}\:\left(\text{peak inspiratory pressure}\right)\right.\\&\left.*\mathrm{RR}\left(\text{respiratory rate}\right)\right]/1000\end{aligned}$$

$$\begin{aligned}\mathrm{VIS}&=\left[\text{Dopamine dose}\left(\mu\mathrm{g}/\mathrm{kg}/\mathrm{min}\right)\right]\\&+\left[\text{Dobutamine dose}\left(\mu\mathrm{g}/\mathrm{kg}/\mathrm{min}\right)\right]\\&+\left[100*\text{Noradrenaline dose}\left(\mu\mathrm{g}/\mathrm{kg}/\mathrm{min}\right)\right]\\&+\left[100*\text{Adrenaline dose}\left(\mu\mathrm{g}/\mathrm{kg}/\mathrm{min}\right)\right]\\&+ \left[10000*\text{Vasopressin dose}\left(\mathrm{U}/\mathrm{kg}/\mathrm{min}\right)\right]\\&+\left[10*\text{Milrinone dose}\left(\mu\mathrm{g}/\mathrm{kg}/\mathrm{min}\right) \right] \end{aligned}$$

For the estimation of the cardiac output, we used the aimed full ECMO flow as described in [31] (Suppl. Table 1), although this represents just an approximation of the resting cardiac output.

### Clinical decision algorithm for ECCO_2_R-CRRT initiation

ECCO₂R was considered in pediatric patients who suffered from ARDS (diagnosis according to the second Pediatric Acute Lung Injury Consensus Conference guidelines) [32] with persistent hypercapnic respiratory failure despite optimized conventional management who required CRRT because of AKI-associated clinically relevant fluid overload complicating respiratory management. Physiological findings prompting consideration of ECCO₂R included persistent hypercapnia (PaCO₂ >60 mmHg), respiratory acidosis (pH < 7.25), and inability to achieve lung-protective ventilation (e.g., PIP > 30 mbar and/or ΔP > 15 mbar). These parameters served as clinical guidance rather than absolute eligibility criteria.

The decision to initiate ECCO₂R was made on an individual basis following multidisciplinary discussion between pediatric intensivists, pediatric nephrologists, and the responsible disease-specific specialists (e.g., pediatric oncologists or pediatric neurologists). Eligibility for vvECMO was assessed independently by a dedicated multidisciplinary ECMO team comprising pediatric intensivists, pediatric cardiologists, ECMO perfusionists, and the respective disease-specific specialists. Decisions regarding vvECMO candidacy considered the patient’s overall prognosis, underlying comorbidities, anticipated reversibility of respiratory failure, and the expected balance of risks and potential benefits. In all included patients, vvECMO was initially considered inappropriate following multidisciplinary assessment.

### Intervention

**Fig. 1:**
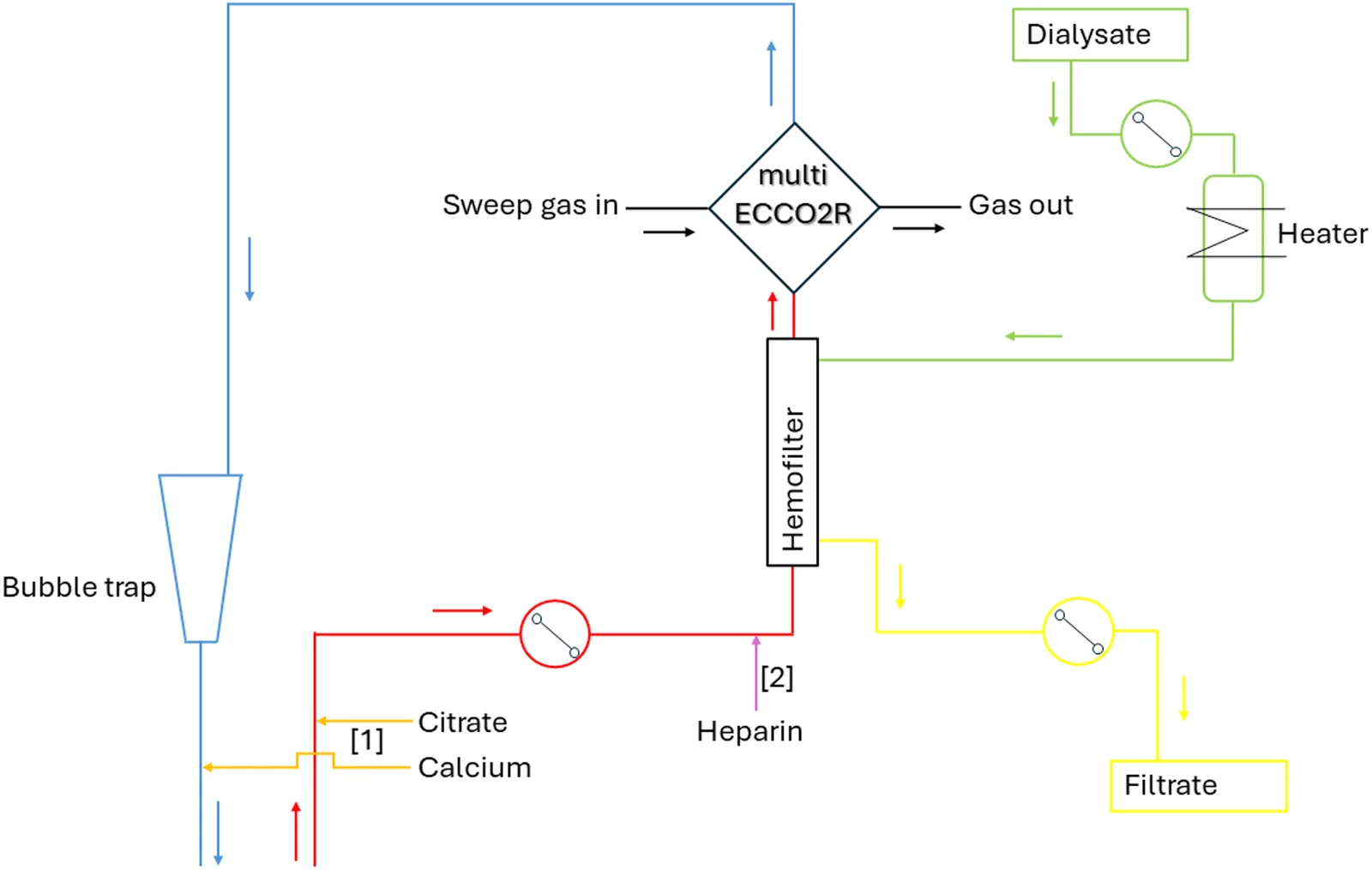
Schematic representation of the ECCO_2_R circuit in combination with CRRT. ECCO_2_R-CRRT may be performed with regional citrate anticoagulation [1] or systemic heparinization [2]. Adapted from Husain-Syed et al. [33] under the Creative Commons Attribution (CC BY) License.

The multiECCO_2_R blood gas exchanger features a membrane lung surface area of 1.35 m^2^ and the priming volume is 100 ml. It can be integrated into a CRRT platform downstream of the hemofilter, facilitating the simultaneous treatment of patients with ARDS and acute kidney injury (ECCO_2_R-CRRT) (Fig. 1).

This licensed medical device is intended for extracorporeal carbon dioxide removal and is designed for supporting the management of patients with respiratory dysfunction in combination with CRRT using a single extracorporeal circuit (ECCO_2_R-CRRT) thereby limiting invasiveness. According to our local practice CRRT was started after establishing a double lumen vascular dialysis cannula (Shaldon catheter). Anticoagulation for CRRT was either performed systemically with unfractioned heparin or regional by using a trisodium citrate solution. Patients were treated in the continuous venovenous hemodialysis (CVVHD) setting using the multiFiltrate device (Fresenius Medical Care, Bad Homburg, Germany).

### Circuit priming and extracorporeal volume

The CRRT circuit was primed according to our institutional standard operating procedure (SOP). The total extracorporeal circuit volume (ECV) was calculated as the sum of the CRRT circuit volume and the 100 mL priming volume of the multiECCO₂R membrane. Estimated circulating blood volume (CBV) was calculated according to age using 80 mL/kg for infants, 75 mL/kg for children, and 70 mL/kg for adolescents, consistent with current pediatric extracorporeal support practice and our SOP. Packed red blood cells were used for circuit priming whenever the total ECV exceeded approximately 10% of the estimated circulating blood volume or when patients exhibited clinically relevant hemodynamic instability requiring vasoactive support before circuit connection. During circuit initiation, patients underwent continuous hemodynamic monitoring.

Although the device is approved only for patients weighing > 40 kg, all treatments were performed in a tertiary pediatric center with extensive experience in extracorporeal therapies. Particular attention was paid to vascular access, circuit pressures, maintenance of adequate blood flow, and continuous hemodynamic monitoring throughout therapy. No patient developed clinically significant hemodynamic instability during circuit initiation.

### Anticoagulation

Prior to initiation of ECCO₂R-CRRT, all patients underwent individualized assessment of bleeding risk during multidisciplinary discussion. Factors considered included active bleeding, thrombocytopenia, coagulation abnormalities, hepatic dysfunction, recent surgery, and the anticipated extracorporeal blood flow required for effective ECCO₂ removal.

Regional citrate anticoagulation was the preferred anticoagulation strategy for isolated CRRT whenever clinically feasible. However, during combined ECCO₂R-CRRT, systemic unfractionated heparin (UFH) was generally preferred, as effective ECCO₂R frequently required blood flows exceeding those compatible with regional citrate anticoagulation. In our institutional CRRT protocol, regional citrate anticoagulation is limited to blood flows of approximately 150–200 mL/min, consistent with the manufacturer’s recommendation, which specifies a maximum blood flow of 200 mL/min during citrate anticoagulation because higher blood flows increase systemic citrate delivery and the risk of citrate accumulation and limit safe application of the combined ECCO₂R-CRRT system [27]. Furthermore, citrate anticoagulation was avoided in patients with impaired hepatic citrate metabolism because of the increased risk of systemic citrate accumulation.

Regional citrate anticoagulation was therefore reserved for selected patients in whom the required extracorporeal blood flow was compatible with citrate anticoagulation and no contraindications existed.

Systemic anticoagulation with UFH was monitored using anti-factor Xa (anti-Xa) activity, targeting 0.3–0.5 U/mL. This therapeutic range was adapted from the recommendations by Giraud et al. [14], who reported a target anti-Xa activity of 0.3–0.4 U/mL. The upper limit was extended to 0.5 U/mL in our institutional protocol to improve the practical management of anticoagulation in critically ill children, reducing the need for frequent anticoagulation adjustments and repeated blood sampling while maintaining effective circuit anticoagulation.

Monitoring of UFH was performed using anti-Xa activity rather than activated partial thromboplastin time (aPTT). Anti-Xa monitoring is an accepted approach for therapeutic UFH monitoring in pediatric patients; the American College of Chest Physicians guideline recommends titrating therapeutic UFH in children to an anti-Xa target of 0.35–0.70 IU/mL, or alternatively to an aPTT range validated against this anti-Xa range. In our critically ill pediatric population, anti-Xa monitoring was preferred because aPTT may be influenced by non-heparin-related alterations in coagulation factor levels and acute-phase responses, potentially resulting in clinically relevant discordance between aPTT and the anticoagulant effect of UFH. Anti-Xa activity provides a more direct assessment of UFH-mediated factor Xa inhibition and was therefore considered more appropriate for UFH dose adjustment in this setting [34–36].

Throughout ECCO₂R-CRRT, laboratory parameters relevant to anticoagulation and hemostasis were monitored regularly in addition to anti-Xa activity. Institutional treatment targets comprised a hemoglobin concentration > 8 g/dL, platelet count > 50 × 10⁹/L, and fibrinogen concentration > 100 mg/dL. Transfusion therapy and anticoagulation were adjusted accordingly to maintain these predefined thresholds.

### ECCO₂R weaning

Weaning from ECCO₂R was considered once lung-protective mechanical ventilation could be maintained with a PIP < 25 mbar, ∆*P* < 14 mbar, PEEP < 10 mbar and stable gas exchange (pH > 7.30 and PaCO₂ <60 mmHg) without increasing ventilatory support. Sweep gas flow was subsequently reduced in 1–2 L/min increments to zero while maintaining the ventilator settings unchanged. If respiratory and hemodynamic stability was maintained for 12–24 h after complete discontinuation of sweep gas flow, the ECCO₂R membrane was removed. This institutional weaning strategy was adapted from the published European ECCO₂R consensus recommendations [37].

### Assessment and definition of adverse events

Adverse events were defined as clinically relevant events occurring after initiation of ECCO₂R-CRRT that were considered by the investigators to be probably related to the extracorporeal support system. Major bleeding was defined as newly occurring clinically significant bleeding requiring therapeutic intervention, transfusion, surgical or endoscopic treatment, or involving a critical anatomical site (e.g., intracranial, gastrointestinal, pulmonary, or vascular access-site bleeding). Clinically apparent hemolysis was defined as hemolysis requiring clinical attention and supported by laboratory findings, including significant increase in bilirubin and lactate dehydrogenase (LDH). Thromboembolic events were defined as newly diagnosed clinically relevant arterial or venous thrombosis occurring after ECCO₂R-CRRT initiation and considered probably attributable to the extracorporeal circuit. New infections were defined as bloodstream or dialysis catheter-associated infections diagnosed after ECCO₂R-CRRT initiation. Device-related complications included circuit clotting requiring premature circuit exchange, membrane failure, or any technical malfunction resulting in interruption of therapy.

Electronic medical records were independently reviewed by one senior consultant in pediatric intensive care medicine and one pediatric nephrologist, who systematically assessed all patient records for predefined adverse events.

### Statistical analysis

Statistical analyses were performed at the patient level, including only the first ECCO₂R-CRRT treatment episode for each pediatric patient to avoid non-independence arising from repeated treatment episodes.

The predefined principal physiological outcomes were PaCO₂, PIP, ΔP, and Vt. Changes in arterial pH, PEEP, OI, VI and VIS were considered exploratory physiological outcomes. Laboratory parameters associated with bleeding and hemolysis, as well as red blood cell and platelet transfusion requirements, were analyzed as safety outcomes.

Repeated measurements of the principal and exploratory physiological outcomes were analyzed using the Friedman test. Laboratory parameters and transfusion requirements, for which baseline and 24-hour measurements were available, were analyzed using the Wilcoxon matched-pairs signed-rank test.

Continuous variables are presented as median and interquartile range (IQR). For the predefined principal physiological outcomes, the median absolute change from baseline to 24 h is additionally reported to facilitate assessment of the magnitude of physiological changes. Given the retrospective design, small sample size, and descriptive nature of this case series, emphasis was placed on the magnitude and direction of observed physiological changes rather than on nominal statistical significance alone. Accordingly, all statistical analyses should be interpreted as exploratory and hypothesis-generating.

No formal sample size calculation was performed because of the retrospective observational study design. Statistical analyses were performed using GraphPad Prism version 10.6.1 (GraphPad Software, Boston, MA, USA). All statistical tests were two-sided, and *P* values < 0.05 were considered statistically significant.

## Results

### Study population

Between November 2023 and November 2025, our institution performed a total of 11 ECCO₂R-CRRT treatment episodes in eight patients, corresponding to 141 cumulative treatment days. One 42-year-old patient with a syndromal disorder who received 6 days of ECCO₂R-CRRT at our pediatric intensive care unit because of severe neurodevelopmental impairment, a body weight of 17 kg, and pediatric body habitus was excluded from the present analysis to maintain a homogeneous pediatric study population.

The final study cohort consisted of the first ECCO₂R-CRRT treatment episode of seven pediatric patients. Complete physiological measurements were available for all patients at each predefined study time point; therefore, no imputation of missing data was required.

The median age of the patients was 12 (IQR 6–14) years and the median weight 24 (IQR 13–65) kg. The median ECCO_2_R duration was 4 (IQR 3–35) days with a median RRT blood flow of 270 (IQR 100–300) ml/min corresponding to 9% (IQR 6–20%) of the estimated cardiac output.

Baseline demographic, clinical characteristics, kidney function parameters as well as ECCO_2_R-CRRT characteristics of the study population are summarized in Tables 1, 2 and 3.

**Table 1:** Baseline demographic and clinical characteristics of the pediatric patients included in the study. Underlying conditions are reported in a generalized manner to preserve patient confidentiality

*P* t.	Age [y]	BBW [kg]	Underlying condition	PARDS severity (OI)	Number of runs	Duration of runs [days]	Survival to PICU discharge
1	14	40	Hematologic disorder after HCT with chronic pulmonary GvHD/bronchiolitis obliterans	moderate (8.8)	3	2	no (changed to palliative setting after weaning from last ECCO_2_R run)
						6	
						50*	
2	12	24	Hematologic malignancy after HCT with TA-TMA	severe (25.3)	1	3	yes
3	12	65	Chronic neurodevelopmental disorder with bacterial pneumonia	severe (49.4)	1	4	yes
4	0.5	9	Immune dysregulation syndrome with DAH	severe (44)	1	2	no (died on vvECMO)
5	7	20	Hematologic disorder after HCT with infectious complication	severe (54.7)	1	3	no (died with ECCO_2_R)
6	15	72	Hematologic malignancy after HCT with infectious complication	severe (28.7)	1	35	no (died with ECCO_2_R)
7	6	13	Hematologic disorder after HCT with chronic pulmonary GvHD/bronchiolitis obliterans	mild (5.1)	2	7	no (weaned from first run, died with ECCO_2_R during second run)
						23	

*BBW*  baseline body weight, *HCT*  hematopoietic cell transplantation, *DAH*  diffuse alveolar hemorrhage, *GvHD*  Graft versus Host Disease, *TA-TMA*  Transplant-associated thrombotic microangiopathy, *PARDS*  Pediatric Acute Respiratory Distress Syndrome, *OI*  Oxygenation Index

* ECCO₂R treatment episode during non-invasive ventilation

**Table 2:** Baseline kidney function, timing of acute kidney injury (AKI), fluid overload (FO), and indications for continuous renal replacement therapy (CRRT). Negative values indicate that AKI had already been diagnosed before PICU admission. AKI was classified according to KDIGO criteria. Fluid overload was calculated from cumulative fluid balance relative to baseline body weight

*P* t.	Time PICU admission to AKI diagnosis [days]	Cr_BASE_ [mg/dl]	Cr_AKI_ [mg/dl]	KDIGO stage at time of AKI diagnosis	Urine output 24 h before CRRT [ml/kg/h]	Cr_CRRT_ [mg/dl]	Fluid overload [%]	AKI duration before CRRT [days]	Indication for CRRT
1	33	0.2	0.44	2	2	0.33	15	21	FO despite diuretics
2	-2	0.63	1.72	2	1	2.12	11	3	Hyperphosphatemia, FO despite diuretics
3	0	0.41	0.73	1	2	0.73	11	1	FO despite diuretics
4	2	0.17	0.43	2	1.75	0.2	18	5	FO despite diuretics
5	2	0.11	0.3	2	2.4	0.28	11	1	FO despite diuretics
6	-3	0.76	3.22	3	0.3	2.13	18	78	Hyperphosphatemia, FO despite diuretics
7	-75	0.2	0.76	3	1	0.1	23	76	FO despite diuretics

*Cr*_BASE_ baseline serum creatinine, *Cr*_AKI_ serum creatinine at AKI diagnosis, *Cr*_CRRT_ serum creatinine at CRRT initiation

**Table 3:** Run-level technical characteristics, complications, and outcome of the first ECCO₂R-CRRT treatment episode. Only the first ECCO₂R-CRRT treatment episode per patient was analyzed to avoid non-independence resulting from repeated treatment episodes. Adverse events refer to new events likely attributed to implementation of ECCO_2_R-CRRT

*P* t.	ECCO_2_*R* BF [ml/min)	Estimated CO [ml/min]	ECCO_2_*R* BF/CO [%]	ECCO_2_*R* GF [l/min]	Shaldon site	Shaldon size [French]	Anti-coagulation	Bleeding event	Thromb-embolic event	Dialysis catheter-associated infection	Hemolysis	Circuit clotting requiring device replacement	ECCO₂*R* discontinuation
1	300	3200	9	4.5	FV	13	Heparin	0	0	0	0	1	succesfully weaned
2	200	2400	8	3.0	FV	11.5	Citrate	0	0	0	0	0	succesfully weaned
3	300	5200	6	4.5	FV	11.5	Heparin	0	0	0	0	0	succesfully weaned
4	100	1125	9	1.5	FV	8	Heparin	0	0	0	0	0	escalated to vvECMO
5	400	2000	20	6.0	FV	11.5	Heparin	0	0	0	0	0	death during ECCO₂R
6	100	5760	2	1.5	FV	11.5	Citrate	0	0	0	0	0	death during ECCO₂R
7	270	1300	21	4.0	FV	11.5	Heparin	0	0	0	0	1	succesfully weaned from first run

*FV*  femoral vein, *BF*  blood flow, *GF*  gas flow, *CO*  cardiac output

All seven patients required invasive mechanical ventilation, CRRT, and ECCO₂R because lung-protective ventilation could not be achieved despite optimized conventional management. Five of seven patients had an underlying hematologic disease following hematopoietic cell transplantation. Five patients presented with severe PARDS, one with moderate PARDS, and one with mild PARDS at ECCO₂R initiation.

All patients fulfilled KDIGO criteria for AKI prior to CRRT initiation and required renal replacement therapy primarily because of clinically relevant fluid overload despite diuretic therapy; two patients additionally required CRRT because of severe hyperphosphatemia (Table 2). Based on serum creatinine criteria alone, one patient fulfilled KDIGO stage 1 AKI, whereas the remaining patients fulfilled stage 2 or 3 AKI. Nevertheless, because KDIGO defines initiation of kidney replacement therapy as stage 3 AKI, all patients met the criteria for KDIGO stage 3 AKI.

Blood flow during the first ECCO₂R-CRRT treatment episode ranged from 100 to 400 mL/min, with sweep gas flows between 1.5 and 6 L/min (Table 3). Systemic unfractionated heparin was used in five patients and regional citrate anticoagulation in two patients. Two treatment episodes required membrane and circuit exchange because of circuit clotting. No bleeding events, thromboembolic complications, clinically apparent hemolysis, or device-related infections attributable to ECCO₂R-CRRT were observed.

All patients underwent ultrasound-guided single-site femoral venous cannulation using an 8-, 11.5-, or 13-French Shaldon catheter (GamCath/Vantive Health Germany, Unterschleißheim, Germany or Joline Medical Technology, Hechingen, Germany).

At baseline, the median arterial pH was 7.30 (IQR 7.21–7.40), the median PaCO₂ was 68 (IQR 59–109) mmHg, the median PIP was 40 (IQR 34–40) mbar, and the median ΔP was 27 (IQR 22–40) mbar. The median baseline VI and OI were 71 (IQR 47–176) and 29 (IQR 9–49), respectively, while the median VIS was 4 (IQR 0–28).

### Principal physiological outcomes

**Fig. 2:**
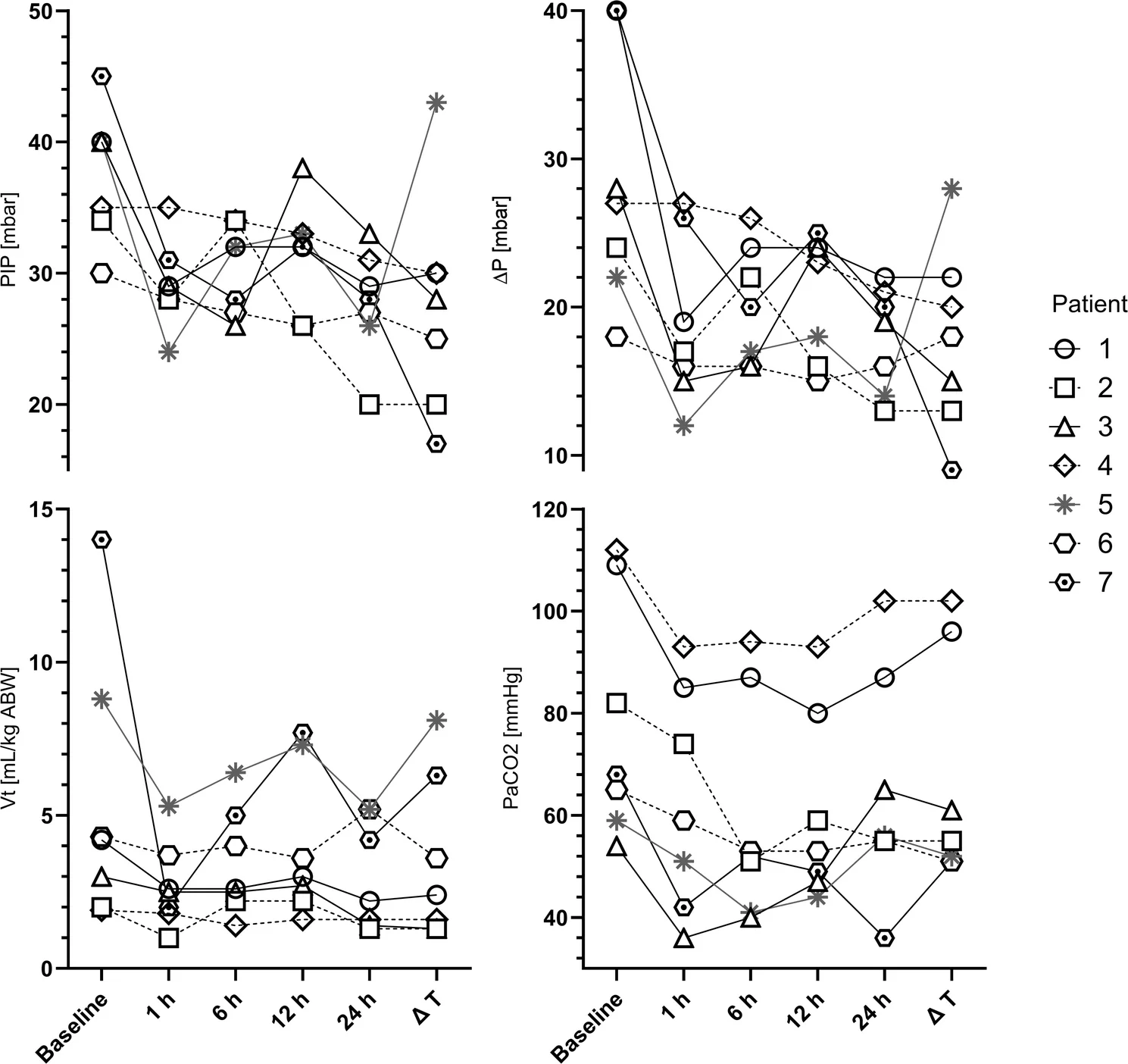
Individual patient trajectories of the primary outcomes during the first ECCO₂R-CRRT treatment episode. Data are shown at baseline, 1, 6 ± 1 h, 12 ± 2 h, and 24 ± 2 h after initiation of ECCO₂R-CRRT, and at the final assessment (ΔT), defined as 7 days after initiation, or within 24 h before discontinuation of ECCO_2_R, if therapy was shorter than 7 days

All predefined principal physiological outcomes demonstrated consistent improvement during ECCO₂R-CRRT (Fig. 2). Friedman analysis showed significant longitudinal changes in PaCO₂, PIP, ΔP, and Vt over the predefined observation period (all *P* < 0.05) (Table 4).

The median absolute changes from baseline to 24 h were − 10 mmHg (IQR − 27 to − 3) for PaCO₂, − 11 mbar (IQR − 14 to − 4) for PIP, − 9 mbar (IQR − 18 to − 6) for ΔP, and − 1.6 mL/kg (IQR − 3.6 to − 0.3) for Vt.

One patient (Patient 5) experienced progressive worsening of ARDS (because of severe diffuse alveolar hemorrhage) despite initial physiological improvement during the first 24 h of ECCO₂R-CRRT. Following repeated multidisciplinary reassessment and at the explicit request of the patient’s caregivers, treatment was escalated to vvECMO. The patient subsequently died from progression of the underlying disease. Despite this individual clinical course, improvement of the predefined principal physiological outcomes remained evident across the study cohort (Fig. 2).

Notably, one patient (Patient 1) underwent prolonged ECCO₂R support during non-invasive ventilation following successful liberation from invasive mechanical ventilation (Table 1).

### Exploratory physiological outcomes

Among the exploratory physiological outcomes, the ventilation index decreased significantly over time (Table 4). In contrast, arterial pH, PEEP, OI, and VIS remained stable throughout the observation period without statistically significant longitudinal changes.

**Table 4:** Time course of the primary and exploratory outcome variables (Baseline, 1, 6 ± 1 h, 12 ± 2 h, and 24 ± 2 h, ∆T) for the first ECCO₂R-CRRT treatment episode of each patient (*n* = 7). Seven observations were available at each predefined study time point. Data are presented as median (IQR)

Variable	Baseline	1-h	6-h	12-h	24-h	∆T	*p* (Friedman)
*Primary outcomes*							
PIP, mbar	40 (34–40)	29 (28–31)	32 (27–34)	32 (26–33)	28 (26–31)	28 (20–30)	***0.007***
∆P, mbar	27 (22–40)	17 (15–26)	20 (16–24)	23 (16–24)	19 (14–21)	18 (13–22)	***0.015***
Vt, ml/kg ABW	4.2 (2-8.8)	2.5 (1.8–3.7)	2.6 (2.2-5.0)	3.0 (2.2–7.3)	2.2 (1.4–5.2)	2.4 (1.3–6.3)	***0.013***
PaCO_2_, mmHg	68 (59–109)	59 (42–85)	52 (41–87)	53 (47–80)	56 (55–87)	55 (51–96)	***0.009***
*Exploratory outcomes*							
pH	7.30 (7.21–7.40)	7.41 (7.23–7.5)	7.40 (7.28–7.43)	7.38 (7.33–7.47)	7.35 (7.29–7.40)	7.36 (7.33–7.41)	0.291
PEEP, mbar	10 (5–12)	11 (8–12)	10 (8–12)	10 (8–14)	10 (7–12)	8 (7–13)	0.945
Oxygenation Index	29 (9–49)	24 (14–34)	29 (12–35)	17 (13–47)	17 (13–37)	14 (9–27)	0.421
Ventilation Index	71 (47–176)	46 (27–131)	42 (37–109)	57 (33–97)	61 (39–97)	69 (26–77)	**0.022**
VIS	4 (0–28)	4 (0–32)	8 (0–32)	7.3 (0–20)	4 (0–30)	7.3 (0–17)	0.616

PIP = peak inspiratory pressure; ∆P = driving pressure; Vt = tidal volume; ABW = actual body weight; PEEP = positive end-expiratory pressure; VIS = vasoactive inotropic score

Bold values indicate statistical significance (*p* < 0.05)

### Safety outcomes

Laboratory parameters associated with bleeding and hemolysis remained stable during the first 24 h of ECCO₂R-CRRT (Table 5). No statistically significant changes were observed in hemoglobin concentration, platelet count, LDH, or total bilirubin (all *P* > 0.05). Similarly, daily red blood cell and platelet transfusion requirements were comparable before and during ECCO₂R-CRRT (both *P* > 0.05).

**Table 5:** Safety outcomes including laboratory values at baseline and 24 h after ECCO_2_R-CRRT initiation, as well as platelet and red blood cell (RBC) transfusions per day – pre and post initiation of ECCO_2_R-CRRT. Data are presented as median (IQR)

Variable	Baseline	24-h	*p* (Wilcoxon)
Hemoglobin, g/dl	9.8 (9-14.4)	11 (9.9–14.4)	0.219
Platelets, G/l	96 (77–191)	81 (72–149)	0.156
LDH, U/l	652 (268–890)	523 (306–707)	0.938
Bilirubin, mg/dl	0.61 (0.38-6)	0.58 (0.42–5.1)	0.469
	**Pre ECCO**_**2**_ **R-CRRT**	**Post ECCO**_**2**_ **R-CRRT**	
RBC transfusions/day	0.5 (0-0.71)	0 (0-0.71)	0.813
Platelet transfusions/day	0.5 (0–1)	0.5 (0-2.4)	0.750

*LDH*  lactate dehydrogenase

Bold values indicate statistical significance (*p* < 0.05)

## Discussion

In this retrospective single-center pediatric case series, integration of ECCO₂R into an already indicated CRRT circuit was associated with rapid reductions in PaCO₂ and ventilatory pressures, thereby facilitating lung-protective mechanical ventilation in critically ill children with combined PARDS and AKI. Four of seven patients were successfully weaned from ECCO₂R, whereas one patient ultimately required vvECMO because of progressive respiratory failure. Although circuit clotting occurred in two of seven analyzed treatment episodes, no major bleeding, clinically apparent hemolysis, thromboembolic complications, or device-related infections considered attributable to ECCO₂R-CRRT were identified. Given the uncontrolled retrospective design, the small sample size, and the concurrent application of CRRT, ventilator adjustments, fluid management, and other intensive care interventions, these findings should be interpreted as descriptive, exploratory, and hypothesis-generating rather than as evidence of treatment efficacy or device safety.

The predefined principal physiological outcomes demonstrated their greatest changes during the first 1–24 h after ECCO₂R initiation. This temporal pattern is consistent with the expected physiological mechanism of extracorporeal carbon dioxide removal and with previous adult studies demonstrating rapid reductions in PaCO₂ following ECCO₂R initiation [11–13]. Nevertheless, the retrospective design does not permit attribution of these physiological changes to ECCO₂R alone. Ventilator settings, fluid removal by CRRT, optimization of medical therapy, and the natural evolution of the underlying disease all occurred concurrently. Accordingly, the observed reductions in PaCO₂, peak inspiratory pressure, driving pressure, tidal volume, and ventilation index should be interpreted as temporal associations rather than proof of a causal treatment effect. At the same time, the early onset of the physiological response within the first hours after ECCO₂R initiation appears more consistent with the immediate effects of extracorporeal carbon dioxide removal than with the slower physiological consequences of CRRT-related fluid management [33, 38, 39].

Despite significant reductions in PaCO₂, arterial pH remained stable throughout treatment. This finding was expected, as bicarbonate-based CVVHD simultaneously corrected metabolic acid-base disturbances while our ventilation strategy continued to allow permissive hypercapnia. Similarly, oxygenation variables did not change significantly, which is physiologically plausible because the multiECCO₂R membrane provides selective carbon dioxide removal rather than clinically relevant oxygen transfer. In contrast, the ventilation index decreased significantly, reflecting the combined reduction in ventilatory pressures and PaCO₂.

The magnitude and direction of the observed physiological changes are consistent with the adult ECCO₂R literature, which demonstrates reductions in PaCO₂ and ventilatory pressures, thereby facilitating lung-protective or ultraprotective ventilation, although without convincing evidence for improved survival [11–13]. Our observations extend these physiological findings to a highly selected pediatric population in whom ECCO₂R was integrated into an already existing CRRT circuit. Because all patients already required renal replacement therapy owing to AKI complicated by clinically relevant fluid overload, this strategy avoided additional vascular access and utilized an extracorporeal circuit that was already clinically indicated. Although the present study cannot determine whether this integrated approach is superior to standalone ECCO₂R, its principal practical advantage lies in minimizing additional extracorporeal hardware in patients already requiring CRRT.

An important aspect of this case series is the institutional clinical decision-making process. ECCO₂R was considered only in highly selected patients who had already fulfilled indications for CRRT and who remained unable to achieve lung-protective ventilation despite optimized conventional management, including persistent hypercapnia, respiratory acidosis, and elevated inspiratory or driving pressures. Importantly, ECCO₂R was not considered a substitute for vvECMO. Decisions regarding ECCO₂R initiation were made jointly by pediatric intensivists, pediatric nephrologists, and disease-specific specialists, whereas candidacy for vvECMO was assessed independently by a multidisciplinary ECMO team comprising pediatric intensivists, pediatric cardiologists, ECMO perfusionists, and the respective disease-specific specialists, as recommended [17]. In all included patients, vvECMO was initially considered inappropriate. The single patient who ultimately underwent vvECMO illustrates the dynamic nature of this decision-making process. Despite initial physiological improvement during the first 24 h of ECCO₂R, progressive deterioration of ARDS prompted repeated multidisciplinary reassessment. At the explicit request of the patient’s caregivers, treatment was escalated to vvECMO; however, the patient subsequently died because of progression of the underlying condition. This clinical course emphasizes that ECCO₂R facilitates carbon dioxide removal but does not alter the progression of severe lung injury or replace established indications for vvECMO. The off-label application of the multiECCO₂R membrane in children weighing below the approved threshold of 40 kg required careful individual risk-benefit assessment. The relatively large membrane surface area and extracorporeal circuit volume raise theoretical concerns regarding hemodynamic instability, coagulation disturbances, hemolysis, and increased transfusion requirements, particularly in smaller children [40, 41]. These considerations were discussed before every treatment during multidisciplinary decision-making. Because of these concerns, we predefined clinically relevant adverse events before data collection and systematically reviewed the complete electronic medical records of all patients. This review was performed independently by one senior pediatric intensivist and one pediatric nephrologist and included clinical documentation, laboratory markers of bleeding and hemolysis, transfusion requirements, and circuit-related complications. Within the limitations of this small retrospective series, no major bleeding events, clinically apparent hemolysis, thromboembolic complications, or device-related infections considered attributable to ECCO₂R-CRRT were identified.

Interestingly, unlike adult randomized trials, our cohort experienced no clinically relevant bleeding events, and no patient required therapy discontinuation due to hemorrhagic complications. This contrasts with adult trials like REST [13], SUPERNOVA [12] and a study by Del Sorbo et al. [42], in which bleeding represented the predominant serious adverse event. On the other hand, adult studies using CRRT platforms likewise did not report relevant adverse events [33, 38, 43].

These observations should not be interpreted as establishing device safety but rather as describing our institutional experience with careful patient selection, intensive monitoring, and defined safety assessment during off-label application. However, premature circuit clotting represented the most frequent device-related complication in our cohort and occurred exclusively during treatment episodes using systemic unfractionated heparin. This observation is consistent with the pediatric CRRT literature, in which regional citrate anticoagulation has repeatedly been associated with prolonged circuit survival and lower rates of filter clotting compared with systemic heparin. Importantly, however, available pediatric evidence has not consistently demonstrated a reduction in clinically relevant bleeding with regional citrate anticoagulation despite its superior circuit patency [44, 45]. In our institution, systemic unfractionated heparin was therefore preferred for most ECCO₂R-CRRT treatments because effective extracorporeal carbon dioxide removal frequently required blood flows exceeding those recommended for regional citrate anticoagulation by the manufacturer, and citrate was additionally avoided in patients at risk of impaired citrate metabolism. Although circuit clotting is clearly undesirable because it interrupts therapy, necessitates circuit exchange, results in blood loss, and may increase transfusion requirements [44, 45], its clinical implications differ fundamentally from thrombosis during ECMO. Unlike ECMO circuit thrombosis, which may directly compromise extracorporeal life support [46], filter clotting during CRRT can usually be managed by timely circuit replacement without immediate danger to the patient. Nevertheless, minimizing premature circuit clotting remains an important objective, and future pediatric studies should evaluate whether modified regional citrate protocols or alternative anticoagulation strategies can safely provide the higher extracorporeal blood flows required for integrated ECCO₂R-CRRT.

Overall mortality was high in this cohort. Six of seven patients presented with moderate or severe PARDS, five had undergone hematopoietic cell transplantation, and six had hematologic or immune-related disorders associated with substantial underlying disease burden. Four patients were successfully weaned from ECCO₂R during the analyzed treatment episode; however, progression of the underlying condition was documented during the subsequent clinical course in all non-survivors. Following detailed review of the complete medical records, no death was attributed to a predefined ECCO₂R-related complication.

Three patients died with refractory respiratory and multiorgan failure secondary to hematopoietic cell transplantation-related pulmonary complications while receiving ECCO₂R-CRRT. One patient died after escalation to vvECMO in the setting of progressive diffuse alveolar hemorrhage resulting in severe PARDS, and one patient was transitioned to palliative care after progression of pulmonary graft-versus-host disease.

Our first patient illustrates the potential clinical role of this strategy. Across three ECCO₂R treatment episodes, ECCO₂R facilitated transition from invasive mechanical ventilation to non-invasive respiratory support despite persistent respiratory insufficiency, thereby reducing exposure to potentially injurious mechanical ventilation. Although this patient was ultimately transitioned to palliative care because of progression of pulmonary graft-versus-host disease, the clinical course exemplifies how integrated ECCO₂R-CRRT may provide meaningful physiological support even when the underlying disease remains irreversible.

Although these clinical courses and documented causes of deterioration provide important context, the uncontrolled retrospective design does not permit attribution of mortality to the underlying diseases or exclusion of a contribution from ECCO₂R-CRRT. The observed mortality should also be interpreted in the context of the high baseline mortality reported for comparable pediatric populations. Mortality among pediatric patients with hematologic or immune-related diseases complicated by (moderate to severe) ARDS has been reported to approach 66% [47], while HCT recipients requiring invasive mechanical ventilation have reported mortality rates of approximately 47% in moderate PARDS and up to 75% in severe PARDS [48]. These data provide important clinical context for the observed mortality but do not permit conclusions regarding the relative contribution of underlying disease severity or ECCO₂R-CRRT to clinical outcome.

This study has several strengths. To our knowledge, it represents the largest reported pediatric case series of ECCO₂R integrated into an already indicated CRRT circuit and provides a detailed description of patient selection, technical implementation, anticoagulation management, weaning, physiological changes, and predefined adverse events. Adverse events were systematically assessed by detailed review of the complete medical records by two experienced physicians, and the analyses were deliberately restricted to the first treatment episode of each patient to avoid bias from repeated observations. Finally, the integrated ECCO₂R-CRRT approach illustrates the close interdisciplinary collaboration between pediatric intensive care, pediatric nephrology, and extracorporeal support teams, highlighting the potential relevance of this strategy across multiple pediatric subspecialties.

Economic aspects were beyond the scope of the present study and were not formally evaluated. Nevertheless, integration of ECCO₂R into an already indicated CRRT circuit avoids the need for an additional extracorporeal platform and vascular access, which may reduce equipment requirements and procedural complexity compared with standalone ECCO₂R or vvECMO. Whether these potential technical advantages translate into meaningful reductions in healthcare costs or resource utilization should be addressed in future prospective studies incorporating formal health-economic analyses.

## Limitations

This study has several limitations. First, the retrospective, uncontrolled, single-center design, the absence of a comparator group, and the small sample size substantially limit statistical precision, preclude causal inference, and prevent definitive conclusions regarding treatment efficacy, clinical benefit, or safety. Accordingly, the present study should be regarded as a descriptive, hypothesis-generating case series rather than an evaluation of treatment effectiveness.

Second, although ECCO₂R-CRRT was temporally associated with rapid reductions in PaCO₂ and ventilatory pressures, the independent physiological contribution of ECCO₂R cannot be separated from concomitant CRRT, ventilator adjustments, fluid management, and other supportive intensive care interventions. Moreover, ventilator adjustments were performed according to clinical judgment rather than a predefined study protocol. Accordingly, the relative contribution of ECCO₂R to the observed physiological changes cannot be quantified.

Third, exact extracorporeal carbon dioxide removal rates were not routinely measured in this pragmatic clinical setting. Therefore, the physiological effect of ECCO₂R was inferred from changes in arterial PaCO₂ and ventilatory parameters rather than direct quantification of membrane CO₂ clearance. Furthermore, biomarkers of ventilator-induced lung injury and advanced assessments of regional lung mechanics, such as electrical impedance tomography or computed tomography, were not performed. Consequently, although the observed reductions in ventilatory pressures are consistent with lung-protective ventilation, the biological impact on ventilator-induced lung injury remains speculative.

Fourth, circuit clotting requiring circuit exchange was the only predefined adverse event identified during systematic review of the complete medical records. Although no major bleeding, clinically apparent hemolysis, thromboembolic complications, or device-related infections were identified, clotting of an integrated ECCO₂R-CRRT circuit necessitates replacement of the complete extracorporeal circuit rather than only the gas-exchange membrane. This may result in treatment interruption, blood loss, increased transfusion requirements, and additional procedural burden, even though its clinical consequences differ fundamentally from circuit thrombosis during ECMO. Further technical refinement of integrated systems and prospective evaluation of anticoagulation strategies and circuit performance are therefore warranted.

Fifth, the study population consisted predominantly of children with hematological malignancies or previous hematopoietic cell transplantation treated at a single tertiary pediatric center with extensive expertise in pediatric extracorporeal support. Consequently, our findings may not be generalizable to other pediatric intensive care populations or institutions with different patient characteristics, resources, or levels of experience.

Finally, use of the multiECCO₂R gas-exchange membrane in children weighing less than 40 kg was outside the manufacturer’s approved labeling. Although no major device-related adverse events beyond those reported were identified during systematic review of the complete medical records, the absence of such events in this small retrospective cohort should not be interpreted as evidence of device safety. Until prospective multicenter studies become available, off-label application of integrated ECCO₂R-CRRT should be reserved for carefully selected patients treated in experienced pediatric centers with expertise in extracorporeal respiratory and renal support.

### Future directions

The present case series raises several clinically relevant questions that warrant prospective investigation. Future multicenter studies should determine whether the physiological improvements associated with integrated ECCO₂R-CRRT translate into clinically meaningful outcomes, including reduced exposure to injurious mechanical ventilation, duration of respiratory support, and survival. In addition, optimal patient selection, timing of ECCO₂R initiation, anticoagulation strategies, circuit design, and standardized weaning protocols remain to be established. Given the rarity and heterogeneity of pediatric patients requiring combined ECCO₂R and CRRT, prospective multicenter observational registries are likely to represent the most appropriate next step to define feasibility, safety, and implementation before comparative interventional studies can reasonably be considered.

## Conclusion

In conclusion, this retrospective pediatric case series suggests that integration of ECCO₂R into an already indicated CRRT circuit is technically feasible and was associated with rapid reductions in PaCO₂ and ventilatory pressures, thereby facilitating lung-protective ventilation in carefully selected children with combined PARDS and AKI. The observed physiological improvements, together with the absence of predefined major device-related complications beyond circuit clotting requiring circuit exchange, support further evaluation of this integrated approach while acknowledging that the present findings remain descriptive and hypothesis-generating rather than evidence of treatment efficacy or device safety. Future prospective multicenter studies should determine whether these physiological benefits translate into clinically meaningful outcomes, better define patient selection, anticoagulation strategies, and standardized weaning protocols, and evaluate how integrated ECCO₂R-CRRT can be optimally incorporated into pediatric extracorporeal respiratory support pathways.

## Supplementary Information

Supplementary Material 1.

Supplementary Material 2.

## Data Availability

The datasets analyzed during the current study are not publicly available due to institutional and data protection regulations but are available from the corresponding author on reasonable request.

## Abbreviations

ABW: Actual body weight
AKI: Acute kidney injury
BBW: Baseline body weight
Cr_AKI_: Serum creatinine at AKI diagnosis
CVVHD: Continuous venovenous hemodialysis
DAH: Diffuse alveolar hemorrhage
∆P: Driving pressure
ECCO_2_R: Extracorporeal carbon dioxide removal
ECMO: Extracorporeal membrane oxygenation
FiO2: Inspiratory oxygen fraction
FO: Fluid overload
GF: Gas flow
HCT: Hematopoietic cell transplantation
IQR: Interquartile range
LDH: Lactat dehydrogenase
OI: Oxygenation index
PaCO_2_: Arterial carbon dioxide tension
PEEP: Positive end-exspiratory pressure
PIP: Peak inspiratory pressure
RBC: Red blood cell
VI: Ventilation index
VIS: Vasoactive inotropic score
Vt: Tidal volume
vv: Venovenous
