# Supplementary material for: Extracorporeal carbon dioxide removal integrated into continuous renal replacement therapy in pediatric patients with ARDS: a retrospective single-center case series
Source: BMC Pediatr. 2026 Sep 9;26:860. doi: 10.1186/s12887-026-07656-y (PMC13560372; doi:10.1186/s12887-026-07656-y)
Supplement: Supplementary file 2 [file 12887_2026_7656_MOESM1_ESM.docx]

***Digital Supplemental Material for the Manuscript – Title Page:***

**Extracorporeal carbon dioxide removal integrated into**

**continuous renal replacement therapy in pediatric patients with ARDS:**

**a retrospective single-center case series**

Martin Kuntz, M.D.^1^; Daniel Matheisl, M.D.^1^; Isabel Schüle, M.D.^2^, Daniel Klotz, Prof.^3^, Hans Fuchs, Prof.^1^, Hendryk Schneider, M.D.^1^

^1^Center for Pediatrics, Division of Neonatology and Pediatric Intensive Care Medicine, Medical Center - University of Freiburg, Faculty of Medicine, University of Freiburg, Freiburg, Germany

^2^Center for Pediatric, Division of Pediatric Nephrology, Medical Center - University of Freiburg, Faculty of Medicine, University of Freiburg, Freiburg, Germany

^3^Bethel Center for Pediatrics, Department of Neonatology and Pediatric Intensive Care Medicine, University Hospital OWL, University of Bielefeld, Germany, Bielefeld, Germany

**Corresponding Author:**

Hendryk Schneider, M.D.,

Center for Pediatrics, Division of Neonatology and Pediatric Intensive Care Medicine,

Medical Center - University of Freiburg, Faculty of Medicine, University of Freiburg,

Breisacher Str. 62, 79106, Freiburg, Germany

Mail: hendryk.schneider@uniklinik-freiburg.de

Tel.: + 49 761 270 43669 ; Fax: + 49 761 270 96 43108

***Table of Contents:***

[**1.**	**Supplemental Table 1**	3](#_Toc233361503)

# 1.  **Supplemental Table 1**

Estimated cardiac output as used for aimed full ECMO flow (from Das S et al., Perfusion 2022; doi: 10.1177/02676591211005260).

| **Body weight (kg)** | **Flow (ml/kg/min)** |
| --- | --- |
| < 7 | 120-200 |
| 7-10 | 100-150 |
| 10-20 | 80-120 |
| 20-25 | 100 |
| >25 | 80 |
